# Ferrostatin-1 alleviates tissue and cell damage in diabetic retinopathy by improving the antioxidant capacity of the Xc^-^-GPX4 system

**DOI:** 10.1038/s41420-022-01141-y

**Published:** 2022-10-25

**Authors:** Jingzhi Shao, Zhouxian Bai, Lirong Zhang, Fengyan Zhang

**Affiliations:** 1 Department of Ophthalmology, the First Affiliated Hospital of Zhengzhou University, Henan Provincial Ophthalmic Hospital, 450052 Zhengzhou, Henan China; 2 Department of Obstetrics and Gynecology, The Genetics and Prenatal Diagnosis Center, The First Affiliated Hospital of Zhengzhou University, 450052 Zhengzhou, Henan China; 3 Department of Pharmacology, School of Basic Medical Sciences, Zhengzhou Unversity, 450000 Zhengzhou, Henan China

**Keywords:** Molecular biology, Drug discovery

## Abstract

Diabetic retinopathy (DR) is a common microvascular complication leading to a high blindness rate among patients with diabetes. Ferroptosis is a type of cell death caused by the accumulation of iron-dependent lipid peroxides. Studies have shown that ferroptosis plays an important role in DR. The rat model of DR was constructed and treated with Ferrostatin-1 (Ferr-1). Haematoxylin and eosin (HE) were used to detect the degree of retinopathy. Oxidative stress levels were detected by ELISA. Perl’s staining was used to detect iron deposition in retinal tissues. Ferritin levels were measured by ELISA. The expression of GPX4 was detected by immunohistochemistry (IHC). GSH/GSSG kit was used to detect the content and proportion of reduced/oxidized glutathione. Western blot was used to detect the expression of ferroptosis-related proteins. TUNEL assay was used to detect cell apoptosis. The expression of GSDMD was detected by fluorescence in situ hybridization (FISH). Western blot was used to detect the expression of apoptosis and pyroptosis-related proteins. Then, high glucose (HG)-induced retinal epithelial cell line ARPE-19 was treated by Erastin (ferroptosis activator) and Ferr-1. CCK-8, ELISA, western blot, flow cytometry, and immunofluorescence (IF) staining were used to detect oxidative stress levels, ferroptosis and cell damage. The mechanism was further explored by adding ferroptosis agonist Erastin. In vitro and in vivo results showed that oxidative stress was increased in DR model, resulting in ferroptosis and tissue or cell damage. After administration of Ferr-1, the antioxidant capacity was improved, ferroptosis levels were reduced and tissue or cell damage was alleviated. In vitro results showed that Ferr-1 reversed the impacts of Erastin on oxidative stress, ferroptosis, and cell damage in HG-induced ARPE-19 cells. Ferr-1 alleviated tissue and cell damage by improving the antioxidant capacity of the Xc^-^-GPX4 system.

## Introduction

Diabetic retinopathy (DR) is the most important manifestation of diabetic microangiopathy, a fundus disease with specific changes [1]. With the improvement of living standards, growing of aging population, and significant changes in lifestyle, the prevalence of DR is increasing. The current study shows that there are 425 million cases of diabetes worldwide and predicts that the number will reach 629 million by 2045 [2]. Notably, DR often causes vision loss or blindness and seriously affects the life quality of thousands of people around the world.

Ferroptosis is a form of cell death caused by the accumulation of iron-dependent lipid peroxides [3]. Ferroptosis pathway is mainly regulated by iron metabolism and oxidative stress. On the one hand, iron metabolism in the organism is tightly regulated for maintaining iron homeostasis. Excessive ferrous ions exert toxic roles and can produce a large number of reactive oxide species (ROS) in the body through the Fenton reaction, which then oxidizes cell membrane lipids and causes ferroptosis [4]. Iron has been reported to be associated with a variety of retinal degenerative diseases, but the in-depth research limits the understanding of the involvement of iron in retinal degenerative diseases. At present, few studies on the direct relationship between DR and ferroptosis are available, but a recent study has confirmed that ferrous ions play a major role in retinal damage, rather than iron ions [5], which is completely in accordance with the ferroptosis mechanism.

On the other hand, ferroptosis is triggered by glutathione (GSH) depletion or glutathione peroxidase 4 (GPx4) inactivation [6, 7]. GSH enables GPx4 to play a central regulatory role in ferroptosis [8]. Through the Cystine/glutamate Antiporter system XC^-^ (System XC^-^), cystine, which is used to synthesize intracellular GSH, is converted from glutamate in a 1:1 ratio [9]. When System XC^-^ was inhibited by compounds such as Erastin and sorafenib, GSH synthesis was reduced, leading to the failure of GPx4 to reduce lipid peroxides using GSH, thus leading to ferroptosis [10]. Therefore, Xc^-^-GPx4 system is an important mechanism for cells to avoid a series of oxidative stress damage caused by lipid peroxides and ferroptosis of cells.

Therefore, this paper will directly explore the ferroptosis phenomenon in DR, and study the underlying regulatory mechanism. Our study may provide additional insights into the mechanisms of ferroptosis in DR and establish a new treatment approach for patients with DR.

## Results

### Ferr-1 ameliorates retinal tissue damage in DR rats

**Fig. 1:**
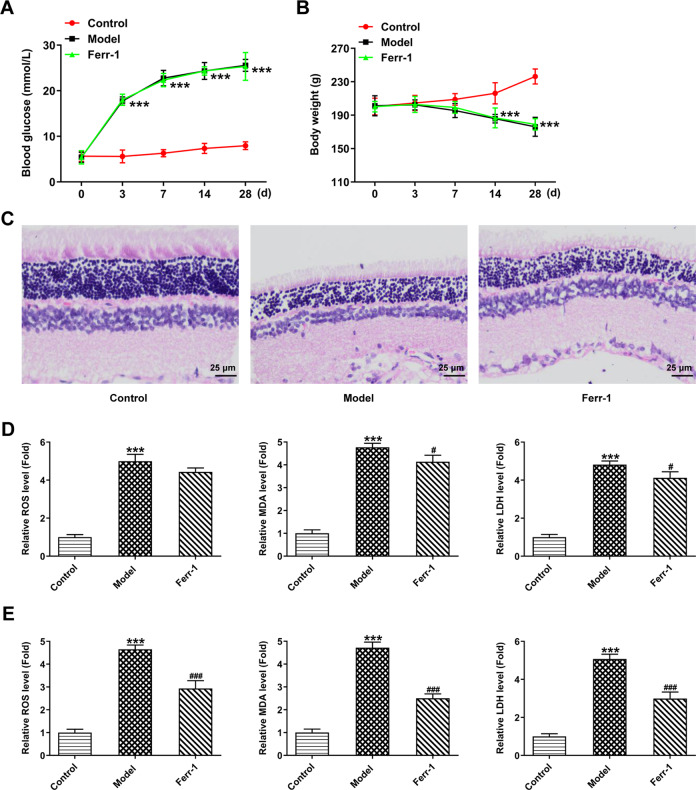
Ferr-1 ameliorates retinal tissue damage in DR rats. **A** Changes in blood glucose in rats. **B** Changes in the body weight of rats. **C** HE detected the degree of retinopathy. The levels of oxidative stress in peripheral blood (**D**) and retinal tissues (**E**) were detected by corresponding kits. ***P < 0.001 vs control. ^#^*P* < 0.05, ^###^*P* < 0.001 vs Model.

Blood glucose and body weight changes in rats were routinely monitored during animal modeling and Ferr-1 intervention. We found that compared with the control group, the blood glucose level of rats in the Model group increased significantly and their body weight decreased significantly. However, there were no significant changes in the Ferr-1 group compared with the Model group (Fig. 1A, B). Pathological results showed that there was no obvious retinal lesion in the control group. In the Model group, the average optical density of inner and outer granular layers decreased, and the distribution of cells was disordered and sparse. Compared with the Model group, the degree of retinopathy in the Ferr-1 group was reduced (Fig. 1C). Then, the levels of oxidative stress in peripheral blood and local retinal tissues were detected by ELISA. The results showed that ROS, MDA, and LDH levels in the Model group were significantly increased compared with the control group. Compared with the Model group, ROS, MDA, and LDH levels, especially in local retinal tissues, were significantly reduced again in the Ferr-1 group (Fig. 1D, E).

### Ferr-1 inhibits ferroptosis in retinal tissues of DR rats

**Fig. 2:**
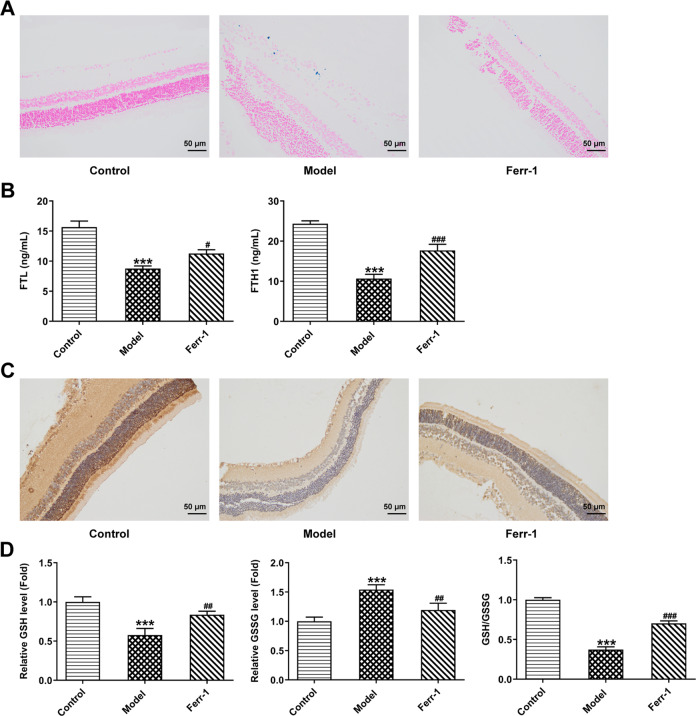
Ferr-1 inhibits ferroptosis in retinal tissues of DR rats. **A** Perl’s staining detected iron deposition in retinal tissues. **B** Ferritin levels in retinal samples were detected by ELISA. **C** IHC detected GPX4 expression level in retinal tissues. **D** GSH/GSSG kit was used to detect the content and proportion of reduced/oxidized glutathione in tissues. ***P < 0.001 vs control. ^#^*P* < 0.05, ^##^*P* < 0.01, ^###^*P* < 0.001 vs Model.

Perl’s staining was used to detect iron deposition in retinal tissues. Compared with the control group, iron deposition occurred in the Model group. Compared with the Model group, the Ferr-1 group showed improved iron deposition (Fig. 2A). The expression of FTL and FTH1 in the Model group was significantly decreased compared with that in the control group, which was reversed after Ferr-1 administration (Fig. 2B). From IHC assay, we found that GPX4 expression was significantly decreased in the Model group compared with the control group. GPX4 expression was increased in the Ferr-1 group compared with the Model group (Fig. 2C). GSH/GSSG activity kit was used to detect the activity level of reduced/oxidized glutathione in retinal tissues. The results showed that compared with the control group, the expression of GSH was decreased, the expression of GSSG was increased, and the ratio of GSH/GSSG was decreased in the Model group. Compared with the Model group, the expression of GSH was increased, the expression of GSSG was decreased, and the ratio of GSH/GSSG was reversed in the Ferr-1 group (Fig. 2D).

**Fig. 3:**
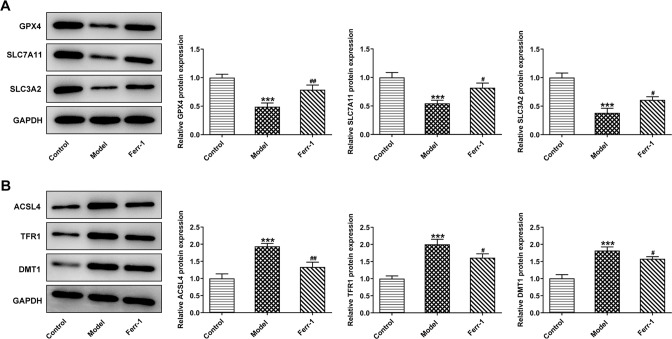
Ferr-1 regulates the expression of ferroptosis associated proteins in retinal tissues of DR rats. **A**, **B** Western blot detected the expression of GPX4, SLC7A11, and SLC3A2. ***P < 0.001 vs control. ^#^*P* < 0.05, ^##^*P* < 0.01, ^###^*P* < 0.001 vs Model.

Western blot was used to detect the expression of ferroptosis-related proteins GPX4, SLC7A11, SLC3A2, ACSL4, TFR1, and DMT1. Compared with the control group, GPX4, SLC7A11, and SLC3A2 expression were decreased while ACSL4, TFR1, and DMT1 expression were increased in Model group. Compared with the Model group, the expression of ferroptosis-related proteins was reversed (Fig. 3A, B). These results indicated that ferroptosis occurred in DR tissues. Ferr-1 significantly inhibited ferroptosis in DR.

### Ferr-1 attenuates cell damage in retinal tissues of DR rats

**Fig. 4:**
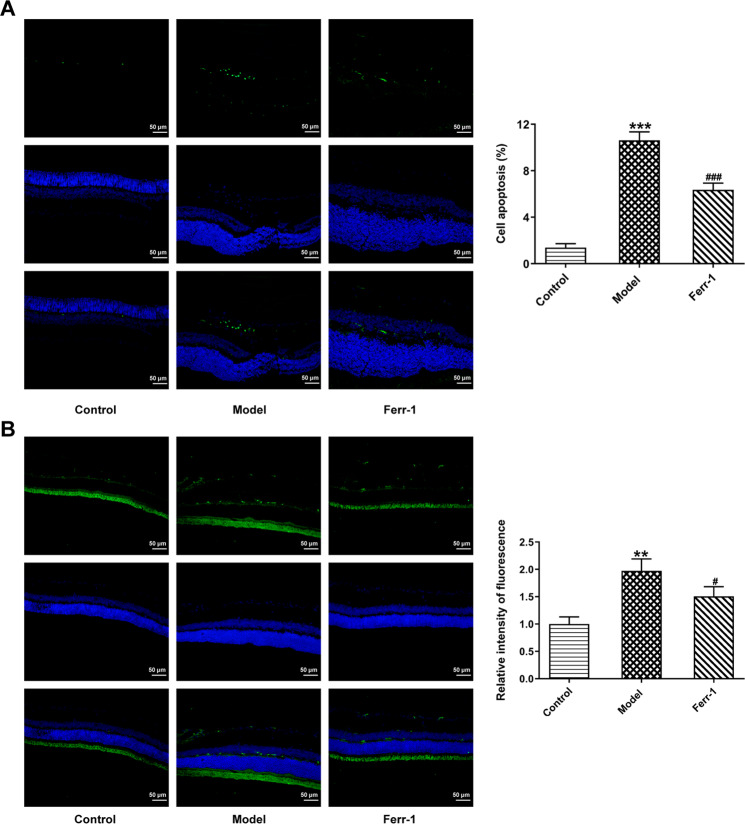
Ferr-1 attenuates cell damage in retinal tissues of DR rats. **A** TUNEL assay detected the cell apoptosis. **B** FISH assay detected the expression of GSDMD in tissues. **P < 0.01, ***P < 0.001 vs control. ^#^*P* < 0.05, ^###^*P* < 0.001 vs Model.

**Fig. 5:**
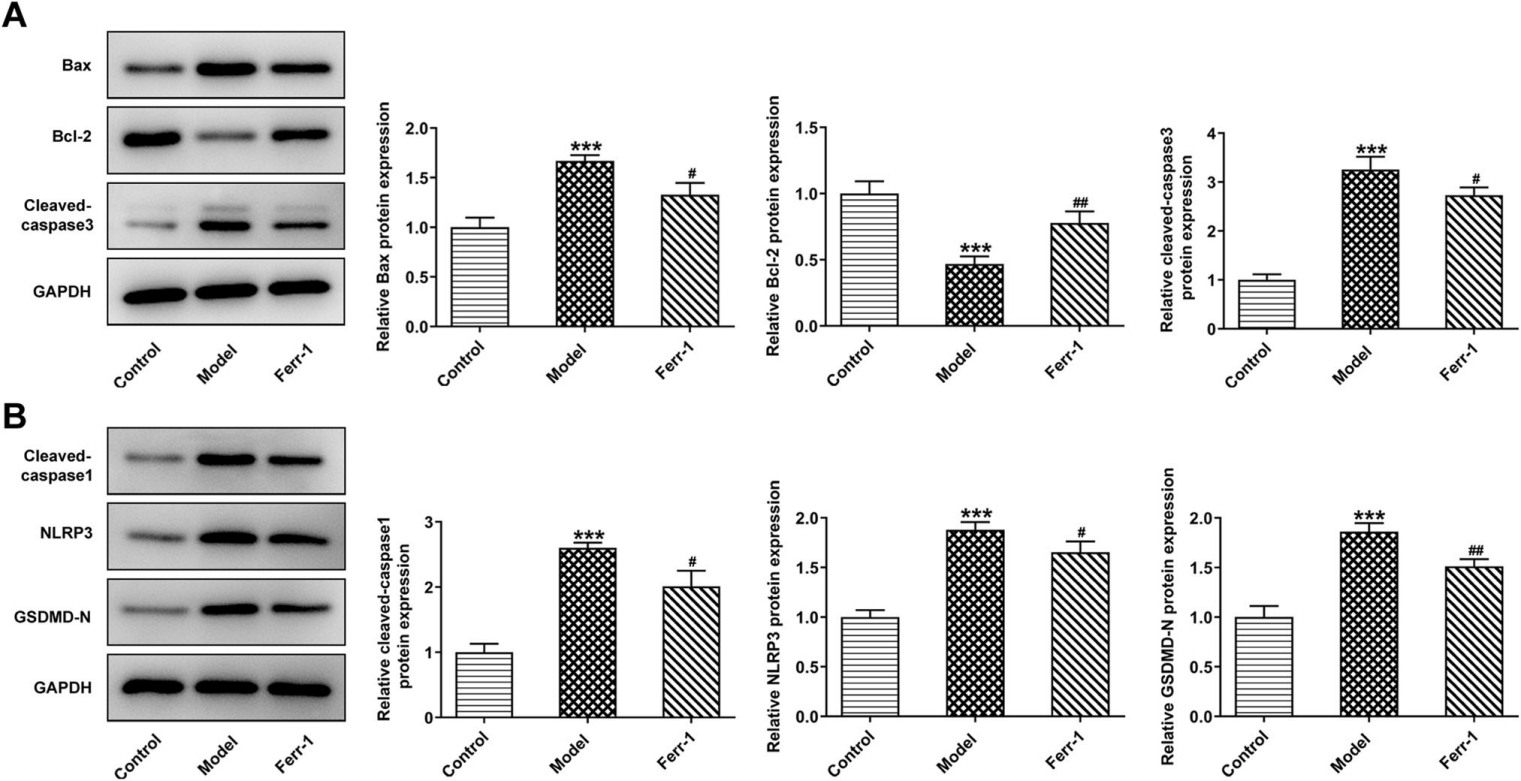
Ferr-1 regulates the expression of apoptosis and pyroptosis-related proteins in retinal tissues of DR rats. **A** Western blot detected the expression of apoptosis-related proteins Bax, Bcl-2, and the cleavage of caspase-3. **B** Western blot detected the expression of pyroptosis-related proteins the cleavage of caspase-1, NLRP3, and GSDMD-N. ***P < 0.001 vs control. ^#^*P* < 0.05, ^##^*P* < 0.01 vs Model.

To detect retinal tissue damage, we measured apoptosis and pyroptosis in retinal tissues. TUNEL results showed that the apoptotic rate of Model group was significantly increased compared with the control group. Compared with the Model group, the apoptotic rate of Ferr-1 group showed a decreasing trend (Fig. 4A). FISH assay was used to detect the expression of GSDMD to detect the level of pyroptosis. The results showed that GSDMD expression in the Model group was significantly increased compared with the control group. Compared with the Model group, the expression of GSDMD in the Ferr-1 group was decreased (Fig. 4B). Western blot analysis of apoptosis and pyroptosis-related proteins showed that compared with the control group, the expression of apoptosis-related proteins Bax was increased, and the cleavage of caspase-3 was increased, and the expression of Bcl-2 was decreased. NLRP3, and GSDMD-N expression and the cleavage of caspase-1 were significantly increased in the Model group. However, the expression of apoptosis and pyroptosis-related proteins was reversed after Ferr-1 administration (Fig. 5A, B). These results indicated that apoptosis and pyroptosis in retinal tissue were significantly exacerbated, and retinal tissues were damaged. Ferr-1 inhibited apoptosis and pyroptosis and ameliorated retinal tissue damage.

### Ferr-1 regulates the Xc^-^-GPX4 system to regulate cell activity and oxidative stress levels in retinal tissues of DR rats

**Fig. 6:**
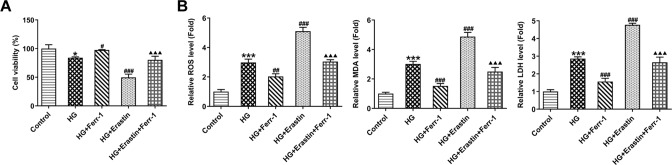
Ferr-1 regulates the Xc^-^-GPX4 system to regulate cell activity and oxidative stress levels in retinal tissues of DR rats. **A** CCK-8 kit was used to detect cell viability. **B** The levels of oxidative stress were detected by corresponding kits. *P < 0.05, ***P < 0.001 vs control. ^#^*P* < 0.05, ^##^*P* < 0.01, ^###^*P* < 0.001 vs HG. ^△△△^*P* < 0.001 vs HG + Erastin.

To further explore the regulatory mechanism of Ferr-1 on DR tissue damage, retinal epithelial cell line ARPE-19 was used for the experiment. High glucose, Erastin (ferroptosis activator) and Ferr-1 intervention were used to treat the cells. CCK-8 kit was used to detect cell activity, and the results showed that the cell activity was decreased significantly after HG induction. Cell activity was increased in the HG + Ferr-1 group and further decreased in the HG + Erastin group compared with the HG group. Cell activity was reversed in the HG + Erastin + Ferr-1 group compared with the HG + Erastin group (Fig. 6A). The results of ROS, MDA, and LDH activity detection showed that compared with the control group, ROS, MDA, and LDH activities were increased in HG group. After Ferr-1 administration, the activities of ROS, MDA, and LDH were decreased again. After Erastin treatment, ROS, MDA, and LDH activities were further increased. ROS, MDA, and LDH expression were inhibited in HG + Erastin+Ferr-1 group compared with HG + Erastin group (Fig. 6B). These results suggested that Ferr-1 regulated the Xc^-^-GPX4 system to regulate cell activity and oxidative stress levels in retinal tissues of DR rats.

### Ferr-1 regulates the redox capacity of the Xc^-^-GPX4 system to regulate ferroptosis in DR cells

**Fig. 7:**
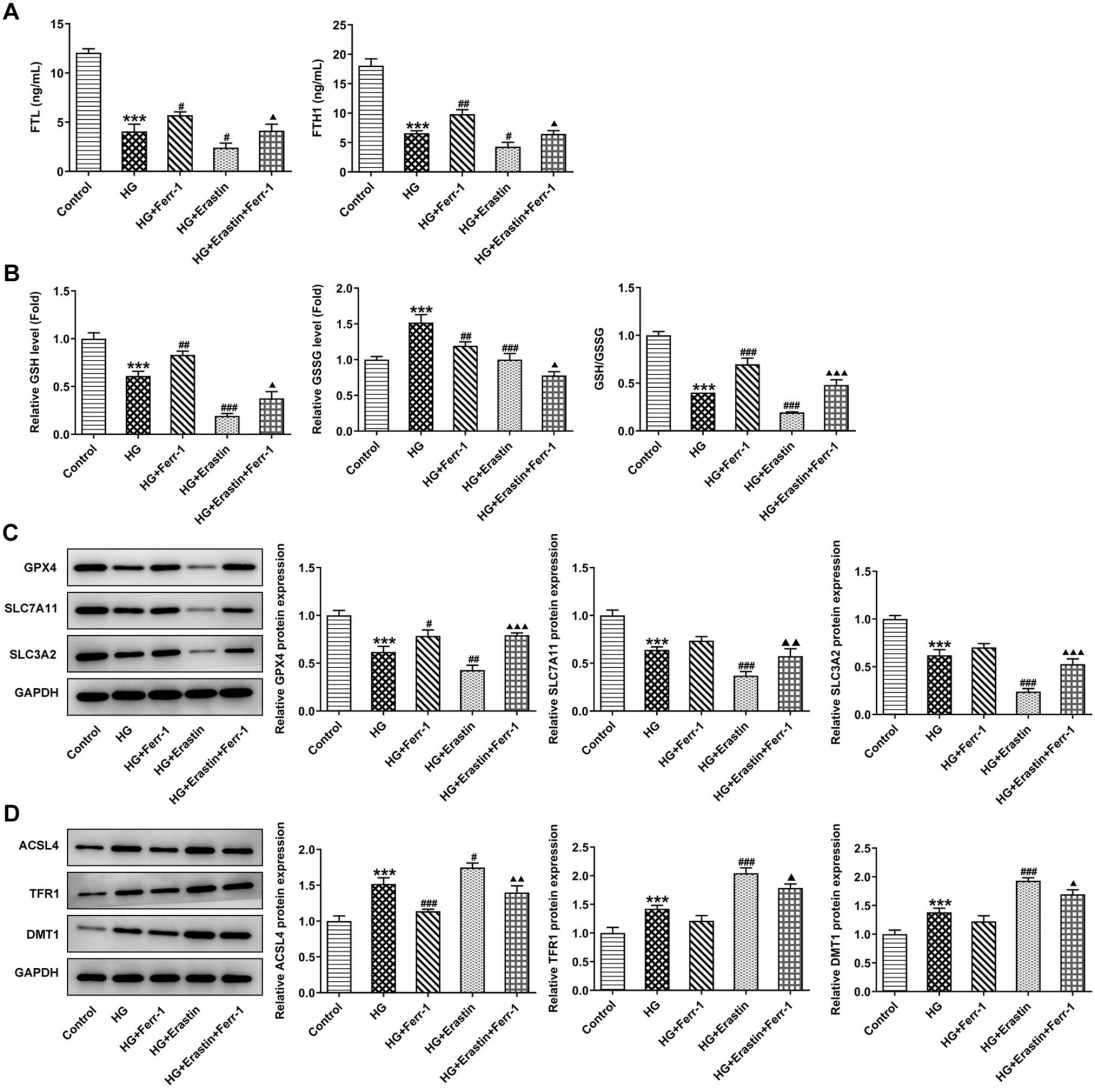
Ferr-1 regulates the redox capacity of the Xc^-^-GPX4 system to regulate ferroptosis in DR cells. **A** Ferritin levels in DR cells were detected by ELISA. **B** GSH/GSSG kit was used to detect the content and proportion of reduced/oxidized glutathione in DR cells. **C**, **D** Western blot detected the expression of ferroptosis-related proteins. ***P < 0.001 vs control. ^#^*P* < 0.05, ^##^*P* < 0.01, ^###^*P* < 0.001 vs HG. ^△^*P* < 0.05, ^△△^*P* < 0.01, ^△△△^*P* < 0.001 vs HG + Erastin.

Ferroptosis in the cells was then measured. Western blot was used to detect the expression of ferritin FTL and FTH1 in cells. The results showed that the expression of FTL and FTH1 were decreased significantly after HG induction. The expression of FTL and FTH1 were increased after Ferr-1 administration and were further inhibited after Erastin administration. Compared with the HG + Erastin group, FTL and FTH1 expression were increased in the HG + Erastin+Ferr-1 group (Fig. 7A). The results of GSH and GSSG showed that compared with the control group, GSH expression was decreased, GSSG expression was increased and the GSH/GSSG ratio was decreased in HG group. After Ferr-1 administration, GSH expression was upregulated, GSSG expression was downregulated, and GSH/GSSG ratio was upregulated. After Erastin treatment, GSH expression was declined, GSSG expression was enhanced, and GSH/GSSG ratio was reduced. Compared with the HG + Erastin+ Ferr-1 group, the expression of GSH was increased, the expression of GSSG was decreased, and the ratio of GSH to GSSG was increased in the HG + Erastin+ Ferr-1 group (Fig. 7B). Western blot results showed that compared with the control group, GPX4, SLC7A11, SLC3A2 expression were decreased in HG group, while ACSL4, TFR1, and DMT1 expression were increased. Compared with HG group, GPX4, SLC7A11, and SLC3A2 were decreased and ACSL4, TFR1, and DMT1 were increased in HG + Ferr-1 group. GPX4, SLC7A11, and SLC3A2 were further decreased and ACSL4, TFR1, and DMT1 were further increased in HG + Erastin group. The expression of Ferroptosis-related proteins were reversed in the HG + Erastin+Ferr-1 group compared with the HG + Erastin group (Fig. 7C, D). These results suggested that Ferr-1 regulated the level of ferroptosis in DR by regulating the redox capacity of the Xc^-^-GPX4 system.

### Ferr-1 regulates the redox capacity of Xc^-^-GPX4 system to inhibit DR cell damage

**Fig. 8:**
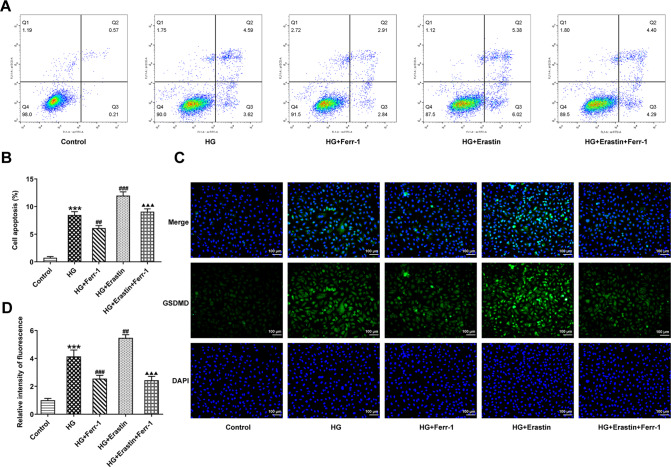
Ferr-1 regulates the redox capacity of Xc^-^-GPX4 system to inhibit DR cell damage. **A** Cell apoptosis was detected by flow cytometry. **B** Statistical diagram of apoptotic cells. **C** IF detected the expression of GSDMD in cells. **D** GSDMD expression statistics. ***P < 0.001 vs control. ^##^*P* < 0.01, ^###^*P* < 0.001 vs HG. ^△△△^*P* < 0.001 vs HG + Erastin.

**Fig. 9:**
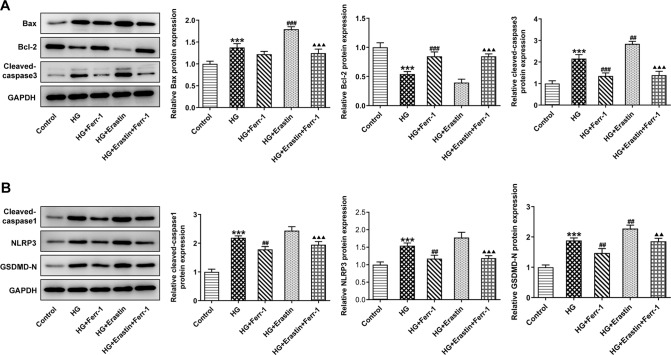
Ferr-1 regulates the expression of apoptosis and pyroptosis-related proteins to inhibit DR cell damage. **A** Western blot detected the expression of apoptosis-related proteins Bax, Bcl-2, and the cleavage of caspase-3. **B** Western blot detected the expression of pyroptosis-related proteins and the cleavage of caspase-1, NLRP3, and GSDMD-N. ***P < 0.001 vs control. ^##^*P* < 0.01, ^###^*P* < 0.001 vs HG. ^△△^*P* < 0.01, ^△△△^*P* < 0.001 vs HG + Erastin.

Apoptosis and pyroptosis levels were then measured. Flow cytometry results showed that the apoptotic rate in HG group was significantly increased compared with the control group and was reduced after Ferr-1 administration. Apoptosis was further increased after Erastin administration. Compared with HG + Erastin group, apoptosis was inhibited in the HG + Erastin+ Ferr-1 group (Fig. 8A, B). IF staining detected the expression of GSDMD in cells, and the results showed that the trend of cell pyroptosis was consistent with cell apoptosis (Fig. 8C, D). Western blot analysis was used to detect Bax, Bcl-2 expression, and the cleavage of caspase-3. We found that Bax expression was increased, the cleavage of caspase-3 was increased and Bcl-2 expression was decreased in the HG + Erastin group compared to HG group. Bax expression was decreased, the cleavage of caspase-3 was decreased and Bcl-2 expression was increased in the HG + Erastin+Ferr-1 group compared with the HG + Erastin group (Fig. 9A). NLRP3 and GSDMD-N expression were increased and the cleavage of caspase-1 was increased in the HG + Erastin group compared to HG group. NLRP3, and GSDMD-N expression were decreased and the cleavage of caspase-1 was decreased in HG + Erastin+ Ferr-1 group compared with HG + Erastin group (Fig. 9B). These results suggested that Ferr-1 regulated cell damage in DR by regulating the redox capacity of Xc^-^-GPX4 system.

## Discussion

System Xc^-^ is a reverse transporter consisting of SLC7A11 and SLC3A2 subunits [11]. More and more studies have proved that inhibition of System Xc^-^ has a key link with ferroptosis. Dixon et al. has established organotypic hippocampal slice culture (OHSC) to infer that the intracellular process of cystine absorption mediated by System Xc^-^ plays an important role in ferroptosis [3]. However, when System Xc^-^ is inhibited, the synthesis of GSH is suppressed, resulting in the failure of GPX4 to reduce lipid peroxides through GSH, and at last leading to ferroptosis [10]. Inhibition of the Xc^-^-GPX4 System results in oxidative damage and even death. A previous study has shown that for retinal cells, GPX4 knockdown directly leads to a significant increase in LDH activity and more apoptosis [12]. In addition, allele variants of the GSH system gene increase the risk of proliferative retinopathy in type 1 diabetics, further demonstrating the essential antioxidant role of GPX4 in retinal cells [13]. In our experiment, it was found that oxidative stress levels in local retinal tissues and peripheral blood of DR rats were significantly increased, while GPX4 expression was decreased, the ratio of GSH/GSSG was decreased, ferroptosis happened in retinal tissues, and retinal tissues were damaged. The ferroptosis inhibitor Ferr-1 alleviated oxidative stress in tissues and thus reduced cell damage. The same results were confirmed by in vitro experiments.

We added Erastin, a ferroptosis activator, to further investigate the mechanism by which Ferr-1 alleviated cell damage. It is known that system Xc^-^ is a reverse transporter consisting of SLC7A11 and SLC3A2 subunits. Erastin is capable of inhibiting the ingestion of SLC7A11, reducing GSH synthesis, and thus inhibiting System Xc^-^, leading to the inactivation of GPX4 and ferroptosis [14]. In this study, in ARPE-19 retinal epithelial cells, Erastin treatment reduced cell viability, increased oxidative stress, decreased GPX4 expression, and increased ferroptosis, resulting in cell damage. These phenomena could be reversed by Ferr-1. That is to say, Ferr-1 could enhance cell viability, reduce oxidative stress level in cells, reverse the expression of GPX4 protein, reduce ferroptosis and reduce cell damage. These results suggested that Ferr-1 could alleviate tissue and cell damage by improving the antioxidant capacity of Xc^-^ -GPX4 system.

Similar to other studies on the antioxidant effect of GSH, the relationship between GSH and GSSG ratio has also been examined to explore the role of GSH in DR. For example, in the study on diabetic retinopathy, the ratio of GSH/GSSG is decreased significantly in the diabetes mellitus group and improved significantly in the distal ischemia group [15]. This suggests that for DR, conventional measures of antioxidant activity are also applicable. Therefore, in our experiment, we detected the expression of GSH, GSSG, and GSH/GSSG ratio to detect the antioxidant performance of tissues and cells. In addition, Erastin inhibited SLC7A11 uptake and reduced GSH synthesis. Ferr-1 did not directly affect SCL7A11, so it was speculated to have little effect on the overall GSH/GSSG ratio. However, Ferr-1 increased GPX4 activity. Therefore, the ratio of GSH to GSSG could be restored to some extent in our study.

We found that compared with the HG group, Ferr-1 significantly downregulated the expression of TFR1 and DMT1 proteins in the retinal tissues of DR rats in animal experiments, but there was no significant changes in TFR1 and DMT1 proteins in in vitro experiments after Ferr-1 treatment in HG-induced ARPE-19 cells. The possible reason is that Ferr-1 can stabilize the intracellular iron pool, but has a limited effect on the uptake and transformation of iron ions, so TFR1 and DMT1, which transport iron into cells and affect the valence state of iron ions, have limited changes. The conjecture needs in-depth study in future experiments.

## Conclusion

In the experiments, we finally proved that Ferr-1 alleviated tissue and cell damage in DR by improving the antioxidant capacity of Xc^-^-GPX4 system. The pathogenesis of ferroptosis in DR was discussed in this paper. And this study might provide a basis for the treatment with ferroptosis inhibitors in DR.

## Materials and methods

### Ethical statement

All of the animal care and experimental procedures were performed according to the Guidelines for the Care and Use of Laboratory Animals, and were approved by the Animal Care and Use Committee of the First Affiliated Hospital of Zhengzhou University (Henan Provincial Ophthalmic Hospital).

### Animal experiments

After SD rats received adaptive feeding for one week, lens was examined by slit lamp microscope before diabetes induction, and lens and corneal defects were excluded. The rats were then divided into Control, Model and Ferrostatin-1 (Ferr-1, ferroptosis inhibitor) groups. Model and Ferr-1 group were given a high-fat and high-sugar diet and intraperitoneal injection of STZ (65 mg/kg body weight) for 4 weeks, which was dissolved in 20 mM sodium citrate buffer and PH 4.5 (10 mg STZ/mL Citrate buffer) after the pH value was adjusted to 7.4. Control group ate a normal diet and was injected with sterile buffer. Glucotrend of Roche was used to detect the glucose content in the blood of each rat during injection. In addition, 5 mg Ferr-1 was dissolved in 5 mL sodium phosphate buffer solution (pH 7.4). The pH of Ferr-1 solution was adjusted to 7.2 before use. Ferr-1 group was given ferr-1 solution for eye drop intervention 5 days before STZ, and the other groups were given artificial tear drops.

### Haematoxylin and eosin (HE) staining

The eyeballs of rats were stained with HE and fixed in 4% buffered paraformaldehyde solution at room temperature for 24 h. Fixed tissues were embedded in paraffin. The sections were subjected to hematoxylin and eosin staining according to the manufacturer’s instructions. The images were visualized and photographed with a light microscope.

### Levels of MDA, LDH, and ROS

MDA and LDH levels in peripheral blood and local retinal tissues were detected using the kit according to the instructions. The intensity was observed by a microplate reader. For ROS, DCFH-DA probes were used for 30 min in the dark. Then, the fluorescence intensity was observed under a fluorescence microplate. The stimulated light wavelength was 485 nm, and the emission light wavelength was 525 nm.

### Perl’s staining

The paraffin slices were placed in a constant temperature box at 60 °C for about 2 h. After dewaxing, the paraffin slices were washed with distilled water for 2 min. Hydrochloric acid and potassium ferrocyanide were mixed equally for 10–20 min before application, followed by phosphate-buffered saline (PBS) washing for 6 min and staining with 3,30-diaminobenzidine (ZSGB-BIO, Beijing, China) for 5 s. An optical microscope was used to observe iron deposition (magnification ×400).

### ELISA

According to the instructions, the expression levels of ferritin FTL (ab155517, Abcam) and FTH1 (ab78877, Abcam) in the retina were detected using the corresponding kits.

### Immunohistochemistry (IHC)

The retinal tissue sections were baked at 60 °C for 1 h and dewaxed in xylene for 30 min. Then, the sections were dehydrated with gradient alcohol of 95, 80, and 75% for 1 min/each, followed by incubation in 3% H_2_O_2_ for 30 min (Beyotime, China) at 37 °C. The sections were placed in 0.01 M citrate buffer, boiled at 95 °C for 20 min, and then allowed to cool down to room temperature. After being sealed in Ultra V Block at room temperature for 5 min, the sections were probed with anti-mouse monoclonal antibody GPX4 (PA5-109274, 1:200, Thermofisher, USA) overnight at 4 °C. Next day, the sections were probed with secondary antibody (SA5-10255, 1:2000, Thermofisher, USA) for 30 min at room temperature. Next, the sections were treated with streptavidin–biotin–peroxidase complex for 30 min and stained with 3 mL of diaminobenzidine (DAB) for 7 min. The sections were subsequently counterstained with hematoxylin, sealed, and fixed with neutral resin. Three areas of each sample in the experiment were randomly captured using a microscope (Leica, Wetzlar, Germany) and analyzed using ImageJ software.

### Glutathione kit (GSH/GSSG)

In all, 0.1 mL reagent was added into retinal tissue homogenate. The mixture was centrifuged for 10 min at 3500 r/min and then the supernatant was removed. The GSH or GSSG levels were respectively detected according to the manufacturer’s protocol (MCE). The absorbance at 420 nm was measured by a microplate reader (Thermofisher, USA).

### Western blot

The protein was extracted and lysed in RIPA buffer (ThermoFisher Scientific, Waltham, USA), and the proteins in these extracts were separated using 12% SDS-PAGE. Then, the separated proteins were transferred to PVDF membranes (ThermoFisher Scientific, Waltham, USA). The membranes were blocked by incubation with 5% skim milk powder for 1.5 h. The membranes were incubated with primary antibodies against GPX4, SLC7A11, SLC3A2, ACSL4, TFR1, DMT1, Bax, Bcl-2, c-caspase-3, c-caspase-1, NLRP3, GSDMD-N and GAPDH (1:1000, Abcam) at 4 °C overnight. Next day, the blot was incubated with a horseradish peroxidase-conjugated secondary antibody (1:5000, Abcam) at room temperature for 1.5 h. Finally, the blots were incubated with ECL reagents (Amersham Pharmacia Biotech, Inc, USA) and visualized using Image J software (GE).

### TUNEL

TUNEL staining was performed using a TUNEL fluorescence kit (Beyotime) to stain the cells. The nuclei of the cells were stained with DAPI (1:5000, Beyotime, China) and determined by calculating the number of cells that showed positive TUNEL staining using a Microscope (SP8, Leica, Japan). Blue fluorescence indicated the nucleus, and green fluorescence indicated positive cells.

### Fluorescence in situ hybridization (FISH)

FISH assay for the GSDMD gene was performed using the DAKO Histology FISH Accessory Kit (Agilent Technologies) according to the manufacturer’s instructions. A 4-µm paraffin-embedded section was cut from whole section blocks. Each prepared section was incubated with 10 μL of the probes, covered with a cover slip and placed in the StatSpin Thermo Brite hybridization system (Abbott Laboratories, Abbott Park, IL, USA) under the following conditions: denatured at 90 °C for 10 min and hybridized at 47 °C for 48 h. After hybridization, the samples were washed with saline sodium phosphate-EDTA (Invitrogen, Grand Island, NY, USA) for 5 min at 47 °C and again at 55 °C for 10 min, and dyed with DAPI again. Finally, images were obtained using a fluorescence microscope (Leica, SP8 confocal laser microscope).

### Cell culture

Retinal epithelial cell line ARPE-19 was purchased from Beina Biological Technology Co., LTD (Shanghai, China) and cultured in DMEM (Biological Industries) supplemented with 10% fetal bovine serum (FBS, Biological Industries). The cells were maintained at 37 °C with 5% CO_2_ and 95% air. High glucose (25.5 mM), Erastin (ferroptosis activator, 10 μM), and Ferr-1 (60 mM) intervention were used to induce the cells and the cells were divided into control group, HG group, HG + Ferr-1 group, HG + Erastin group, and HG + Erastin+ Ferr-1 group.

### CCK-8

Cell viability was assessed using counting kit-8 (CCK-8) (Dojindo Molecular Technologies, Inc., Tokyo, Japan) according to the manufacturer’s protocol. After High glucose, Erastin and Ferr-1 intervention were used to treat the cells, the cells were incubated with the CCK-8 solution for 2 h, and then the cell viability was measured at the absorbance of 450 nm using a microplate reader (Molecular Devices, Sunnyvale, CA, USA).

### Flow cytometry

ARPE-19 cells (1 × 10^5^ cells/mL) were grown in DMEM with 10% FBS for 12 h on six‐well plates. Then, the cells were treated with High glucose, Erastin, and Ferr-1, and the treated cells were harvested. The cells were resuspended in binding buffer and stained with 5 μL Annexin V‐FITC plus 5 μL propidium iodide (Beyotime, Shanghai, China) at room temperature for 30 min in the dark. The binding buffer was added to stain cells and washed three times to remove excess dyes, and then resuspended in 500 μL binding buffer. The percentage of apoptotic cells was analyzed using flow cytometry (BD, FACSCalibur, USA).

### Immunofluorescence (IF) staining

ARPE-19 cells (2 × 10^4^ cells/mL) were seeded in 12-well plates. Then, the cells were treated with high glucose, Erastin, and Ferr-1, and the treated cells were harvested. Afterward, the cells were washed with PBS, fixed with paraformaldehyde, permeabilized with methanol, and counterstained with 4′,6-diamidino-2-phenylindole to visualize the nuclei. The cell images were obtained with a fluorescence microscope (EVOS; Life Technologies, Carlsbad, CA, USA).

### Statistical analysis

Statistical significance between two groups was determined with a Student *t* test, and statistical significance among multiple groups was determined with one-way analysis of variance, followed by a Tukey post hoc test using GraphPad Prism software version 8 (GraphPad Software Inc., San Diego, CA). Data were presented as means ± SD. *P* < 0.05 was considered to be statistically significant.

## Supplementary information

Original Data File

## Data Availability

The analyzed datasets generated during this study are available from the corresponding author on reasonable request.
