# Supplementary material for: Ferrostatin-1 alleviates tissue and cell damage in diabetic retinopathy by improving the antioxidant capacity of the Xc--GPX4 system
Source: Cell Death Discov. 2022 Oct 25;8:426. doi: 10.1038/s41420-022-01141-y (PMC9596714; doi:10.1038/s41420-022-01141-y)
Supplement: Supplementary file 1 — Original Data File [file 41420_2022_1141_MOESM1_ESM.docx]

**Fig3A**












**Fig3B**












**Fig5A**

**





**

**

**

**Fig5B**

**





**

**

**

**Fig7C**

**





**

**

**

**Fig7D**

**





**

**

**

**Fig9A**

**





**

**

**

**Fig9B**
